# Virus‐Specific Impact of Respiratory Viruses on Adult Emergency Department Outcomes

**DOI:** 10.1002/jmv.71039

**Published:** 2026-06-26

**Authors:** Daijiro Nabeya, Takeshi Kinjo, Naoya Nishiyama, Wakaki Kami, Wakako Arakaki, Takuma Okamoto, Kenta Kuniyoshi, Mao Nishiyama, Hideta Nakamura, Shusaku Haranaga, Jiro Fujita, Kazuko Yamamoto, Soichi Shiiki, Tomoo Kishaba

**Affiliations:** ^1^ Internal Medicine III (Pulmonary Medicine and Medical Oncology) Wakayama Medical University Graduate School of Medicine Wakayama Japan; ^2^ Department of Infectious, Respiratory and Digestive Medicine, Graduate School of Medicine University of the Ryukyus Okinawa Japan; ^3^ Department of Respiratory Medicine Ohama Dai‐ichi Hospital Okinawa Japan; ^4^ Department of Infectious Diseases Okinawa Chubu Hospital Okinawa Japan; ^5^ Department of Respiratory Medicine Okinawa Chubu Hospital Okinawa Japan

**Keywords:** coinfection, community‐acquired respiratory viruses, emergency department, hospitalization, respiratory tract infections, rhinovirus

## Abstract

Few studies have examined the impact of community‐acquired respiratory viruses (CRVs) on emergency department visits and outcomes in adults. This study aimed to characterize the epidemiology of CRV infections and assess associations between virus types and clinical outcomes. We retrospectively reviewed adult patients with respiratory symptoms who underwent rapid influenza testing at a community hospital emergency department in Okinawa, Japan, from February 2020 to January 2021. Residual samples were tested using multiplex polymerase chain reaction assays to identify CRVs. Among 551 cases, 228 (41%) tested positive for viruses. Compared with virus‐negative patients, there were no significant differences in median age (64 years, range 20–99) or the proportions requiring oxygen supplementation (*n* = 60) or hospitalization (*n* = 125). The common cold was more frequent in virus‐positive patients (*n* = 74, *p* = 0.002). Rhinovirus was the most prevalent virus (*n* = 111) and accounted for the highest numbers of oxygen supplementation (*n* = 26) and hospitalizations (*n* = 61). Seasonal coronavirus was significantly associated with oxygen supplementation (*p* = 0.029), and co‐detection of multiple viruses was associated with hospitalization (*p* = 0.046). These findings highlight the clinical impact of CRVs on adult emergency department outcomes. Point‐of‐care virus diagnostics may improve risk stratification and prediction of outcomes based on virus types and co‐infections.

## 1 Introduction

Community‐acquired respiratory virus (CRV) infections, commonly referred to as cold viruses, excluding influenza and severe acute respiratory syndrome coronavirus 2 (SARS‐CoV‐2), are among the most frequently diagnosed conditions in adults and contribute substantially to the global social, economic, and disease burden [1, 2, 3, 4]. Unlike the temporary outbreaks seen with emerging infections, CRV infections comprise a diverse range of viruses that circulate continuously throughout the year, with their impact persisting from the past to the present and into the future. Currently, no vaccines are available for most CRV types, and the global population will likely continue to face the challenges posed by these infections. To improve patient care in clinical settings and advance vaccine development in basic research, further clinical studies of CRV infections are urgently needed.

Many clinical studies on respiratory virus infections in adults have focused on hospitalized cases of community‐acquired pneumonia [5], major CRV types such as rhinovirus and respiratory syncytial virus [6, 7, 8], or immunocompromised patients [9, 10]. These studies typically discuss outcomes such as disease severity and mortality. However, identifying the most relevant virus types associated with emergency department visits in adults and the virus types most strongly linked to subsequent hospitalization is equally important. Few studies have addressed these aspects, as most research has focused on pediatric populations [11] or specific virus types [8]. This study provides novel insights by analyzing the impact of respiratory virus infections in adult emergency department patients, stratified by virus types.

The aim of this study is to address these gaps by investigating the prevalence, trends, and characteristics of CRV infections among adult patients presenting to the emergency department. Additionally, we aimed to identify which CRV types have the greatest impact on adverse outcomes in this clinical setting. Before the coronavirus disease 2019 (COVID‐19) pandemic, the emergency department of Okinawa Chubu Hospital, a community hospital in Okinawa, routinely performed influenza antigen testing year‐round for patients presenting with acute respiratory symptoms. In this study, we analyzed residual samples from these influenza tests to investigate CRVs among adults who underwent influenza testing in the emergency department.

## 2 Materials and Methods

### 2.1 Study Design and Patient Selection

This was a retrospective study. Adult patients who presented to the emergency department of Okinawa Chubu Hospital between February 2020 and January 2021 and underwent influenza rapid antigen testing (nasal swab) were included. For these patients, medical records were reviewed to identify those with any respiratory symptoms, including rhinitis, sore throat, cough, sputum production, and dyspnea. Cases without documented respiratory symptoms were excluded. For these patients, physician diagnoses at the time of the emergency department visit were recorded. Additional information, including patient background, underlying diseases, and post‐emergency department visit outcomes, was also collected. During this period, the hospital did not perform influenza antigen testing on patients who were clearly suspected of having COVID‐19. Consequently, samples from these patients were not collected for this study.

Based on results of virus testing, patients were categorized based on virus detection status: CRVs group (rhinovirus, respiratory syncytial virus, metapneumovirus, seasonal coronavirus, parainfluenza virus, enterovirus, adenovirus, bocavirus, dual detection of two or more CRVs), influenza virus, SARS‐CoV‐2, and virus‐negative group. Patients with dual detection of a CRV and influenza virus or SARS‐CoV‐2 were classified under the influenza virus/SARS‐CoV‐2 group.

### 2.2 Specimens

The residual fluid from the rapid influenza antigen testing was used as the specimen. The ImunoAce® Flu test kit from Tauns Laboratories (Shizuoka, Japan) was utilized. The specimens were temporarily stored at 4°C, and gene extraction was performed using the QIAamp® MinElute® Virus Spin Kit (QIAGEN, Netherlands) at the University of the Ryukyus laboratory before storage at −80°C. At the time of polymerase chain reaction (PCR) testing, the specimen was thawed and examined using a commercial PCR kit for respiratory viruses (Anyplex™ II RV16 Detection, Seegene, Korea). It is capable of detecting the following community‐acquired respiratory viruses: influenza A virus, influenza B virus, human rhinovirus, respiratory syncytial virus A and B, metapneumovirus, parainfluenza virus 1, 2, 3, and 4, coronavirus 229E, NL63, and OC43, adenovirus, enterovirus, and bocavirus. HCoV‐HKU1 was not included in the assay panel. PCR testing for SARS‐CoV‐2 was conducted using the Anyplex™ 2019‐nCoV Assay (Seegene, Korea).

### 2.3 Study Objectives and Analysis

The study aimed to assess whether CRV infections are associated with post‐emergency department visit outcomes (requiring oxygen supplementation and requiring hospitalization) in adults with respiratory symptoms, analyzed by virus types. It also aimed to determine the proportion of CRV infections among emergency department patients with respiratory symptoms, and to compare patient backgrounds, diagnoses at presentation, and post‐emergency department visit outcomes by CRV types.

The cases were categorized into a CRV‐positive group and a virus‐negative group, and their backgrounds, clinical diagnoses, and outcomes were compared. As reference groups, influenza virus and SARS‐CoV‐2 cases were also included. The CRVs cases were further classified by detected virus types, and the proportions and total counts of viral detections relative to the entire study population were presented. The backgrounds, clinical diagnoses, and outcomes of each CRVs types were compared. Additionally, factors associated with each outcome were statistically analyzed. These analyses aimed to identify which CRV types had the greatest impact on specific outcomes.

### 2.4 Statistical Analysis

For two‐group comparisons, categorical variables were analyzed using the Chi‐square test or Fisher's exact test, and continuous variables were assessed using the *t*‐test or Mann–Whitney *U* test. For comparisons among three or more groups, categorical variables were evaluated using the Chi‐square test or likelihood ratio test, and continuous variables were analyzed using the Kruskal‐Wallis test. Binomial logistic regression was conducted to examine associations between respiratory virus detections and post‐emergency department visit outcomes (oxygen supplementation and hospitalization). Independent variables included background factors potentially associated with each outcome, detection of specific CRV types, detection of any CRV, and co‐detection of two or more CRVs. The model was built using stepwise forward selection (likelihood ratio). All statistical analyses were performed using IBM SPSS Statistics for Windows, Version 22.0 (IBM Corp., Armonk, NY, USA).

## 3 Results

**Figure 1:**
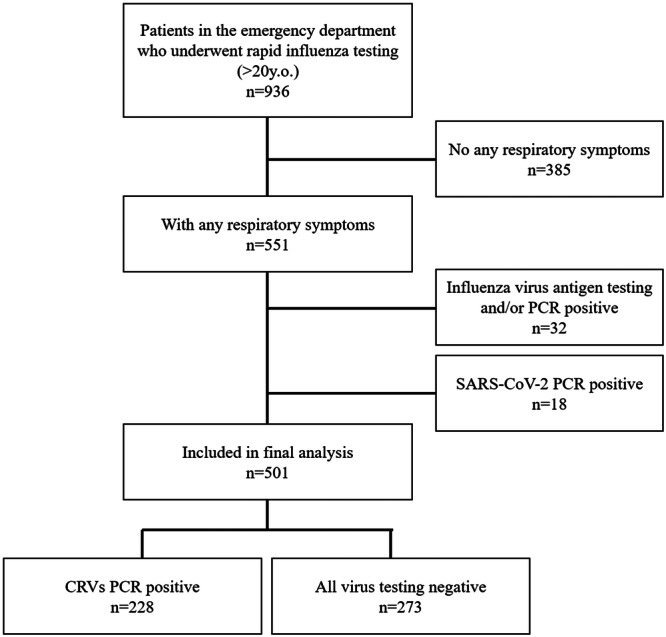
Flowchart. Flowchart of study participant selection. Among 936 adult patients who underwent influenza testing in the emergency department during the study period, 551 patients with respiratory symptoms were identified. After excluding patients positive for influenza or SARS‐CoV‐2, 501 patients were included in the final analysis. Abbreviations: COVID‐19, coronavirus disease 2019; CRV, community‐acquired respiratory virus; PCR, polymerase chain reaction.

**Figure 2:**
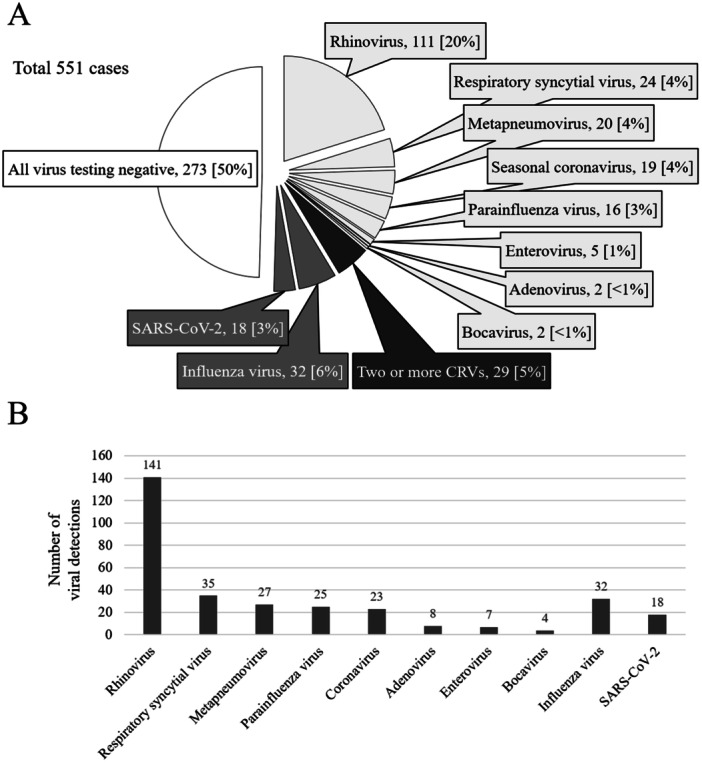
Details of viral detections. A: Distribution of viral detections among 551 adult patients with respiratory symptoms who underwent influenza testing in the emergency department. The pie chart categorizes participants into mono‐detection of CRVs, co‐detection of multiple CRVs, influenza virus detection, SARS‐CoV‐2 detection, and virus‐negative cases. Cases with co‐detection of influenza virus or SARS‐CoV‐2 with CRVs are included in the respective influenza or SARS‐CoV‐2 groups. Overall, viral detection was positive in half of the study population (278/551), with CRVs detected in 228 patients. B: Number of viral detections by virus types. The bar chart shows the number of viral detections for each virus types, with each detected virus counted separately in cases with co‐detections. Abbreviations: CRV, community‐acquired respiratory virus; SARS‐CoV‐2, severe acute respiratory syndrome coronavirus 2.

A total of 936 samples were collected, and 551 cases were included in the study. Of these, 278 cases (50%) tested positive for at least one virus (Figure 1). CRVs were detected in 228 cases (41%), influenza was detected in 32 cases (6%) (28 by rapid antigen test, 28 by PCR, with overlap), and SARS‐CoV‐2 was detected in 18 cases (3%) (the influenza and SARS‐CoV‐2 groups included cases with detection of CRVs). Among study participants by virus detection status, cases of rhinovirus mono‐detections were the most common (111, 20%), followed by detections of two or more CRVs (29, 5%) (Figure 2‐A). Regarding the distribution of detected respiratory viruses by types, rhinovirus was overwhelmingly dominant with 141 cases, while respiratory syncytial virus, metapneumovirus, parainfluenza virus, and seasonal coronaviruses were detected at similar frequencies, each with 20–30 cases (Figure 2‐B). Bocavirus, adenovirus, and enterovirus were detected in only a few cases. These proportions of each virus type are consistent with previous reports in adult populations [5, 12, 13, 14, 15]. A summary of subtype distribution is provided in Suppl 1.

The comparison between the CRV‐positive and virus‐negative groups is shown in Table 1. Patients positive for influenza or SARS‐CoV‐2 were excluded from this analysis (Suppl 2). There were no significant differences in baseline characteristics, underlying conditions, or post‐emergency department visit outcomes. However, as expected, upper respiratory tract infections were more frequently diagnosed in the CRV‐positive group (74 [32%]) than in the CRV‐negative group (56 [21%], *p* = 0.002).

**Table 1:** Patient characteristics among 501 adult patients with respiratory symptoms who underwent influenza testing in the emergency department, excluding patients positive for influenza or SARS‐CoV‐2.

	CRVs detection	All virus test negative	*p*‐value
*N*	228	273	
**Backgrounds**			
Male/female	122/106	142/131	0.739
Age, median (range)	64 (20–99)	68 (20–103)	0.340
Treated with immunosuppressants, *n* (%)	24 (11)	32 (12)	0.672
Utilization of a care facility, *n* (%)	66 (29)	92 (34)	0.254
**Underlying diseases**			
Bronchial asthma, *n* (%)	50 (22)	44 (16)	0.097
Chronic obstructive pulmonry disease, *n* (%)	25 (11)	39 (14)	0.267
Interstitial lung disease, *n* (%)	9 (4)	13 (5)	0.658
Bronchiectasis, *n* (%)	8 (4)	15 (5)	0.290
Chronic heart failure, *n* (%)	35 (15)	43 (16)	0.902
Neurological and neuropsychiatric disorders, *n* (%)	52 (23)	74 (27)	0.269
Malignant diseases, *n* (%)	22 (10)	26 (10)	0.962
End stage chronic kidney diseases, *n* (%)	8 (4)	12 (4)	0.614
Autoimmune diseases, *n* (%)	9 (4)	16 (6)	0.327
Diabetes mellitus, *n* (%)	25 (11)	31 (11)	0.890
**Physician diagnoses in the emergency department**			
Respiratory infectious diseases			
Common colds, *n* (%)	74 (32)	56 (21)	**0.002**
Bacterial pharyngitis, *n* (%)	10 (4)	21 (8)	0.126
Acute bronchitis, *n* (%)	12 (5)	15 (5)	0.909
Bacterial pneumonia, *n* (%)	78 (34)	90 (33)	0.769
Exacerbation of cardiopulmonary diseases			
Bronchial asthma, *n* (%)	12 (5)	9 (3)	0.274
Chronic obstructive pulmonary disease, *n* (%)	8 (4)	9 (3)	0.896
Interstitial lung disease, *n* (%)	2 (1)	7 (3)	0.140
Chronic heart diseases, *n* (%)	9 (4)	10 (4)	0.868
Non‐respiratory infections and others*, *n* (%)	23 (10)	56 (21)	**0.001**
**Post‐emergency department visit outcomes**			
Requiring oxygen supplementation, *n* (%)	60 (26)	72 (26)	0.988
Requiring hospitalization, *n* (%)	125 (55)	151 (55)	0.913
Death within 30 days, *n* (%)	8 (4)	6 (2)	0.375

*Note:* Categorical variables were compared using the Chi‐square test or Fisher's exact test, and continuous variables were assessed using the *t*‐test or Mann–Whitney *U* test.

Abbreviations: CRV, community‐acquired respiratory virus; SARS‐CoV‐2, severe acute respiratory syndrome coronavirus 2.

Table 2 presents a detailed analysis of CRV‐detected patients by virus type, distinguishing between single detection and co‐detection of CRVs. Rhinovirus infection was by far the most common, accounting for the largest numbers of oxygen supplementation (*n* = 26) and hospitalizations (*n* = 61) among CRV‐positive cases. Nevertheless, the proportions within rhinovirus‐positive patients were not the highest (23% and 55%, respectively). In comparison of outcomes, patients with seasonal coronavirus and those with two or more CRVs had higher incidences of oxygen supplementation and hospitalization compared to other CRVs (47% and 74%, 41% and 72%, respectively).

**Table 2:** Patient characteristics according to CRV type among 228 CRV‐positive patients.

	Rhinovirus	Respiratory syncytial virus	Metapneumovirus	Coronavirus	Parainfluenza virus	Enterovirus	Adenovirus	Bocavirus	Two or more CRVs	*p*‐value
*N*	111	24	20	19	16	5	2	2	29	
**Backgrounds**										
Male/female	62/49	12/12	10/10	9/10	7/9	4/1	1/1	1/1	16/13	0.938
Age, median (range)	64 (20–99)	61 (27–91)	60 (20–93)	75 (20–96)	65 (31–98)	30 (23–42)	48 (26–70)	51 (30–71)	66 (24–99)	0.145
Treated with immunosuppressants, *n* (%)	11 (10)	3 (13)	2 (10)	1 (5)	3 (19)	0	0	1 (50)	3 (10)	0.727
Utilization of a care facility, *n* (%)	33 (30)	5 (21)	5 (25)	8 (42)	5 (31)	0	0	0	10 (34)	0.333
**Underlying diseases**										
Bronchial asthma, *n* (%)	25 (23)	5 (21)	4 (20)	6 (32)	5 (31)	0	0	0	5 (17)	0.578
Chronic obstructive pulmonary disease, *n* (%)	13 (12)	0	1 (5)	4 (21)	2 (13)	0	0	1 (50)	4 (14)	0.155
Interstitial lung disease, *n* (%)	3 (3)	2 (8)	0	1 (5)	1 (6)	0	0	0	2 (7)	0.800
Bronchiectasis, *n* (%)	1 (1)	1 (4)	2 (10)	0	2 (13)	0	0	0	2 (7)	0.267
Chronic heart disease, *n* (%)	20 (18)	3 (13)	3 (15)	6 (32)	1 (6)	0	0	0	2 (7)	0.260
Neurological and neuropsychiatric disorders, *n* (%)	23 (21)	5 (21)	5 (25)	7 (37)	2 (16)	0	1 (50)	0	9 (31)	0.364
Malignant diseases, *n* (%)	12 (11)	3 (13)	0	2 (11)	1 (6)	0	0	1 (50)	3 (10)	0.411
End stage chronic kidney diseases, *n* (%)	7 (6)	0	1 (5)	0	0	0	0	0	0	0.311
Autoimmune diseases, *n* (%)	4 (4)	1 (4)	1 (5)	0	2 (13)	0	0	0	1 (3)	0.823
Diabetes mellitus, *n* (%)	11 (10)	5 (21)	1 (5)	5 (26)	0	0	0	0	3 (10)	0.140
**Physician diagnosis in the emergency department**										
Common colds, *n* (%)	37 (33)	9 (38)	7 (35)	4 (21)	5 (31)	3 (60)	1 (50)	1 (50)	7 (24)	0.791
Bacterial pharyngitis, *n* (%)	4 (4)	1 (4)	0	0	1 (6)	2 (40)	0	0	2 (7)	0.227
Acute bronchitis, *n* (%)	4 (4)	0	3 (15)	1 (5)	3 (19)	0	0	0	1 (3)	0.219
Bacterial pneumonia, *n* (%)	35 (32)	5 (21)	7 (35)	8 (42)	6 (38)	0	1 (50)	1 (50)	15 (52)	0.182
Exacerbation of cardiopulmonary diseases, *n* (%)	18 (16)	3 (13)	1 (5)	6 (32)	0	0	0	0	3 (10)	0.085
Non‐respiratory infections and others*, *n* (%)	13 (12)	6 (25)	2 (10)	0	1 (6)	0	0	0	1 (3)	0.120
**Post‐emergency department visit outcomes**										
Requiring oxygen supplementation, *n* (%)	26 (23)	4 (17)	3 (15)	9 (47)	6 (38)	0	0	0	12 (41)	**0.034**
Requiring hospitalization, *n* (%)	61 (55)	12 (50)	8 (40)	14 (74)	7 (44)	0	1 (50)	1 (50)	21 (72)	**0.026**
Death within 30 days, *n* (%)	6 (5)	0	1 (5)	0	0	0	0	0	1 (3)	0.648

*Note:* For comparisons among three or more groups, categorical variables were evaluated using the chi‐square test or likelihood ratio test, and continuous variables were analyzed using the Kruskal–Wallis test.

Abbreviations: CRV, community‐acquired respiratory virus; SARS‐CoV‐2, severe acute respiratory syndrome coronavirus 2.

**Figure 3:**
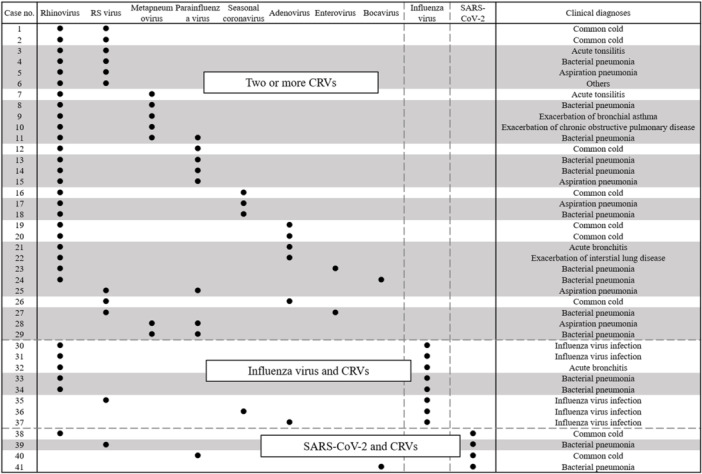
Cases with detection of two or more virus types. The figure shows virus combinations and corresponding diagnoses among cases with detection of two or more virus types. Rhinovirus is the most common virus detected across all cases. Gray‐highlighted items indicate hospitalized cases. The proportion hospitalized was higher in cases with detection of two or more CRVs (21/29, 72%) compared to those with co‐detection involving influenza virus (2/8, 25%) or SARS‐CoV‐2 (1/4, 25%). Abbreviations: CRV, community‐acquired respiratory virus; SARS‐CoV‐2, severe acute respiratory syndrome coronavirus 2.

Figure 3 shows cases in which two or more viruses were detected. No specific combination of viruses was found to be particularly common; however, rhinovirus was the most frequently involved, consistent with the overall virus detection ratio, being identified in 30 of the 41 co‐detection cases. Rhinovirus co‐detections were observed with all other viral types. The proportion requiring hospitalization was higher in cases of detection of two or more CRVs compared to those involving co‐detection with influenza virus or SARS‐CoV‐2.

Table 3 presents the results of a binomial logistic regression analysis to determine the association between respiratory virus detections and post‐emergency department visit outcomes. The analysis included age and other factors potentially related to outcomes, along with the detection of CRV by type. Seasonal coronavirus detection was significantly associated with requiring oxygen supplementation at the time of emergency department presentation. Additionally, detection of two or more CRVs was significantly associated with requiring hospitalization. While the odds ratios for both were close to 3, the 95% confidence intervals were wider compared to other significant factors. Viral detections were not associated with mortality outcomes; instead, immunosuppressive therapy was the only factor linked to mortality. Due to the low overall number of deaths, a robust analysis of this outcome was not possible.

**Table 3:** Multivariable analysis of factors associated with post‐emergency department visit outcomes in adults with respiratory symptoms.

	Odds ratio	95% Confidence interval	*p*‐value
**A: Requiring oxygen supplementation *n* = 138**
Age (years old)	1.046	1.033–1.059	< 0.001
Seasonal coronavirus detections	3.042	1.121–8.254	0.029
Chronic respiratory diseases	2.788	1.782–4.362	< 0.001
Chronic heart diseases	1.978	1.139–3.436	0.015
**B: Requiring hospitalization *n* = 289**			
Age (years old)	1.047	1.035–1.059	< 0.001
Co‐detection of two or more CRVs	2.839	1.021–7.893	0.046
Utilization of a care facility	2.650	1.380–5.087	0.003
Chronic respiratory diseases	2.887	1.814–4.596	< 0.001
Neurological and neuropsychiatric disorders	1.898	1.009–3.571	0.047

*Note:* A: A binary logistic regression analysis was performed using stepwise forward selection (likelihood ratio) with requiring oxygen supplementation in the emergency department as the dependent variable. Variables that were not retained in the final model included individual CRV positivity (each other than seasonal coronavirus), any CRV positivity, SARS‐CoV‐2 PCR positivity, history of malignancy, history of end‐stage renal failure, history of neurological and neuropsychiatric disorders, and immunosuppressive treatment. The overall significance of the model was confirmed by the omnibus test (*p* < 0.05), and model fit was assessed using the Hosmer‐Lemeshow test (*p* > 0.05). The model's overall classification accuracy was 77.5%. B: A binary logistic regression analysis was performed using stepwise forward selection (likelihood ratio) with emergency hospitalization as the dependent variable. Variables that were not retained in the final model included individual CRV positivity, any CRV positivity, SARS‐CoV‐2 PCR positivity, history of chronic heart disease, history of malignancy, history of end‐stage renal failure, history of diabetes mellitus, and immunosuppressive treatment. The overall significance of the model was confirmed by the omnibus test (*p* < 0.05), and model fit was assessed using the Hosmer‐Lemeshow test (*p* > 0.05). The model's overall classification accuracy was 78.9%.

Abbreviations: CRV, community‐acquired respiratory virus; PCR, polymerase chain reaction; SARS‐CoV‐2, severe acute respiratory syndrome coronavirus 2.

## 4 Discussion

This study quantified the prevalence of CRV detections among emergency department patients with suspected airway infections and aimed to identify which CRV type warrant particular attention. The findings demonstrated that seasonal coronavirus detections were significantly associated with requiring oxygen supplementation at emergency department presentation, while the detection of two or more CRVs was significantly associated with hospitalization. Implementing accurate point‐of‐care testing for seasonal coronavirus and CRV co‐infections in the emergency department could further enhance risk stratification and optimize patient management. Although these associations were statistically significant, the absolute number of such cases was relatively small. In contrast, rhinovirus was the most frequently detected virus and accounted for the highest total number of cases requiring oxygen supplementation and hospitalization. This high burden likely reflects its widespread community circulation, posing challenges for targeted interventions at the emergency department level. Nevertheless, public health measures, including infection prevention strategies and the development of a rhinovirus vaccine, may help reduce the overall burden of rhinovirus‐related hospital visits.

Clinical data on seasonal coronavirus infections are limited compared to other major CRVs [16], making it challenging to assess whether their severity is higher, lower, or comparable to that of other viruses. In immunocompromised patients, seasonal coronaviruses can cause lower respiratory tract infections [9], similar to other respiratory viruses in this population [10]. However, no studies have specifically focused on community‐acquired pneumonia caused by seasonal coronaviruses in immunocompetent adults. In this study, the incidence of pneumonia in patients with seasonal coronavirus detections was comparable to that in other virus groups. However, the higher prevalence of cardiopulmonary disease exacerbations in this group likely contributed to the increased frequency of cases requiring oxygen supplementation. Nevertheless, due to the small sample size, further studies in adult populations are needed to validate these findings.

Previous studies have primarily focused on co‐infections involving highly pathogenic respiratory viruses, such as influenza and SARS‐CoV‐2, with CRVs, suggesting that such co‐infections may lead to more severe disease outcomes [17, 18, 19]. While studies in adult populations have not consistently demonstrated an association between detections of two or more CRVs and worse clinical outcomes [20], some pediatric studies have suggested a possible link to increased disease severity [21]. Based on these findings, co‐detection of two or more respiratory viruses may be associated with hospitalization; however, the clinical significance of respiratory viral co‐detection remains uncertain, and these findings should be interpreted cautiously given the inconsistent findings in previous studies. Our study's findings on the potential association between the co‐detection of multiple CRVs and the risk of requiring hospitalization may offer valuable insights, particularly given the year‐round circulation of CRVs in the community.

Rhinovirus was not significantly associated with adverse outcomes in this study; however, its high prevalence makes it a critical concern in the emergency department. Although rhinovirus infections are generally mild [22, 23, 24], they circulate year‐round, affect individuals across all age groups, and have a substantial economic impact [2, 5, 11, 23, 25]. Vaccine development for rhinovirus remains challenging due to its extensive serotype diversity [26, 27]. While public awareness campaigns on infection prevention may have limited effectiveness in the general population, our findings that older age and residence in care facilities were associated with hospitalization suggest that targeted prevention strategies for older adults and care facility residents may be more effective than universal measures.

This study has several limitations. First, this study had several limitations related to patient selection. During the study period, patients suspected of COVID‐19 at the emergency department were excluded from the study and managed in a separate clinical pathway. Most of these patients later tested negative for SARS‐CoV‐2, suggesting that some may have had CRV infections. Notably, CRV cases with symptoms resembling COVID‐19—potentially more severe—may have been excluded, possibly influencing the observed proportions requiring oxygen supplementation and hospitalization. In addition, during the study period, influenza antigen testing was routinely performed year‐round at the study hospital for patients presenting with acute respiratory symptoms because influenza circulation was common throughout the year in Okinawa. Nevertheless, the study population was limited to patients who underwent influenza testing, and selection bias related to testing practices and clinical suspicion could not be completely excluded. This may have influenced the observed proportions of detected viruses, particularly for respiratory viruses that co‐circulate with influenza. Second, this was a single‐center study conducted at the emergency department with a high proportion of older adults visits and care facility residents visits, which may have affected disease severity and the proportion hospitalized. Since CRV infections can be severe in older adults, and viral prevalence varies regionally—even within Japan—our findings may not be generalizable. A multicenter study would provide broader insights. Third, we did not perform direct comparisons between CRVs and influenza or COVID‐19 due to differences in diagnostic approaches and disease severity. Influenza cases were primarily identified by point‐of‐care tests, often in younger, milder patients seeking antiviral therapy. COVID‐19 cases were retrospectively confirmed and did not include patients with severe pneumonia or respiratory failure. These differences, combined with limited point‐of‐care testing for CRVs, complicate direct outcome comparisons. Fourth, interpreting pathogen detection results requires caution. PCR testing has high sensitivity, and positive results do not always indicate active infection. Conversely, some rare viruses not targeted by the assay may have been present in PCR‐negative cases. Contamination or colonization cannot be ruled out even when a virus is detected. Finally, the statistical analysis had limitations. The sample sizes for individual virus type were small, limiting the power to detect associations with clinical outcomes. The use of stepwise selection may introduce overfitting or model instability. Due to the small number of deaths, we could not adequately evaluate 30‐day mortality. Notably, as this study aimed to explore potential associations by virus types, adjustments for multiple comparisons were not performed.

## 5 Conclusions

CRVs were detected in approximately half of adult emergency department patients presenting with respiratory symptoms. Seasonal coronavirus detection was significantly associated with oxygen supplementation at presentation, while the detection of two or more CRVs was significantly associated with hospitalization. However, rhinovirus had the greatest overall impact due to its high prevalence. These findings highlight the role of CRVs in post‐emergency department outcomes. Introducing point‐of‐care diagnostics for CRVs in emergency department patients could clarify the underlying disease process that led to the visit and further aid in predicting post‐visit outcomes based on the detected virus types and the presence of co‐infections.

## Author Contributions

Conceptualization: D. Nabeya, T. Kinjo. Data curation: D. Nabeya, N. Nishiyama, W. Kami, W. Arakaki, T. Okamoto, K. Kuniyoshi, M. Nishiyama. Formal analysis: D. Nabeya, T. Kinjo. Investigation: D. Nabeya, N. Nishiyama, W. Kami, W. Arakaki. Methodology: D. Nabeya, T. Kinjo. Project administration: D. Nabeya, T. Kinjo. Resources: D. Nabeya, N. Nishiyama, W. Kami, W. Arakaki, T. Okamoto, K. Kuniyoshi, M. Nishiyama. Supervision: H. Nakamura, S. Haranaga, J. Fujita, K. Yamamoto, S. Shiiki, T. Kishaba. Validation: H. Nakamura, S. Haranaga, J. Fujita, K. Yamamoto, S. Shiiki, T. Kishaba. Visualization: D. Nabeya. Writing – original draft: D. Nabeya. Writing – review and editing: N. Nishiyama, W. Kami, W. Arakaki, T. Okamoto, K. Kuniyoshi, M. Nishiyama, H. Nakamura, S. Haranaga, J. Fujita, K. Yamamoto, S. Shiiki, T. Kishaba.

## Funding

The authors have nothing to report.

## Ethics Statement

This study was conducted in accordance with the Declaration of Helsinki and the Ethical Guidelines for Medical and Biological Research Involving Human Subjects in Japan. The protocol was approved by the ethics committees of Okinawa Chubu Hospital (2019‐63) and the University of the Ryukyus (R2‐1628). Given its retrospective design and use of anonymized residual specimens, the requirement for individual informed consent was waived, and an opt‐out approach was implemented on the hospital website.

## Conflicts of Interest

The authors declare no conflicts of interest.

## Supporting information

Supporting File 1.

Supporting File 2.

## Data Availability

The datasets generated during this study are not publicly available due to patient privacy restrictions but are available from the corresponding author on reasonable request.
