# Supplementary material for: Virus‐Specific Impact of Respiratory Viruses on Adult Emergency Department Outcomes
Source: J Med Virol. 2026 Jun 26;98(7):e71039. doi: 10.1002/jmv.71039 (PMC13306531; doi:10.1002/jmv.71039)
Supplement: Supplementary file 1 — Supporting File 1. [file JMV-98-e71039-s001.docx]

Suppl 1. Data on the distribution of influenza, respiratory syncytial virus, parainfluenza, and coronavirus subtypes

| Virus | Subtype | n |
| --- | --- | --- |
| Influenza virus | A | 27 |
|  | B | 5 |
| Respiratory syncytial virus | A | 1 |
|  | B | 34 |
| Parainfluenza virus | 1 | 9 |
|  | 2 | 1 |
|  | 3 | 1 |
|  | 4 | 14 |
| Seasonal coronavirus | OC43 | 21 |
|  | NL63 | 2 |
|  | 229E | 0 |
