# Supplementary material for: Virus‐Specific Impact of Respiratory Viruses on Adult Emergency Department Outcomes
Source: J Med Virol. 2026 Jun 26;98(7):e71039. doi: 10.1002/jmv.71039 (PMC13306531; doi:10.1002/jmv.71039)
Supplement: Supplementary file 2 — Supporting File 2. [file JMV-98-e71039-s002.docx]

Suppl 2. Data for cases with detected influenza or SARS-CoV-2 are provided as reference (These cases include co-detections with CRVs.)

|  | Influenza virus detection | SARS-CoV-2 detection |
| --- | --- | --- |
| N | 32 | 18 |
| **Backgrounds** |  |  |
| Male/Female | 13/19 | 12/6 |
| Age, median (range) | 47 (20-87) | 62 (23-91) |
| Treated with immunosuppressants, n (%) | 0 | 0 |
| Utilization of a care facility, n (%) | 3 (9) | 7 (39) |
| **Underlying diseases** |  |  |
| Bronchial asthma, n (%) | 6 (19) | 2 (11) |
| Chronic obstructive pulmonry disease, n (%) | 1 (3) | 2 (11) |
| Interstitial lung disease, n (%) | 0 | 1 (6) |
| Bronchiectasis, n (%) | 0 | 0 |
| Chronic heart failure, n (%) | 2 (6) | 3 (17) |
| Neurological and neuropsychiatric disorders, n (%) | 4 (13) | 6 (33) |
| Malignant diseases, n (%) | 0 | 0 |
| End stage chronic kidney diseases, n (%) | 0 | 0 |
| Autoimmune diseases, n (%) | 0 | 0 |
| Diabetes mellitus, n (%) | 3 (9) | 1 (6) |
| **Physician diagnoses in the emergency department** |  |  |
| Respiratory infectious diseases |  |  |
| Common colds, n (%) | 0 | 3 (17) |
| Influenza virus infection, n (%) | 28 (88) | 0 |
| Bacterial pharyngitis, n (%) | 0 | 4 |
| Acute bronchitis, n (%) | 2 (6) | 3 (17) |
| Bacterial pneumonia, n (%) | 2 (6) | 7 (39) |
| Exacerbation of cardiopulmonary diseases, |  |  |
| Bronchial asthma, n (%) | 0 | 0 |
| Chronic obstructive pulmonary disease, n (%) | 0 | 0 |
| Interstitial lung disease, n (%) | 0 | 1 (6) |
| Chronic heart diseases, n (%) | 0 | 0 |
| Non-respiratory infections and others*, n (%) | 0 | 0 |
| **Post-emergency department visit outcomes** |  |  |
| Requiring oxygen supplementation, n (%) | 2 (6) | 4 (22) |
| Requiring hospitalization, n (%) | 5 (16) | 8 (44) |
| Death within 30 days, n (%) | 0 | 0 |
